# High Thermally Stable and Melt Processable Polyimide Resins Based on Phenylethynyl-Terminated Oligoimides Containing Siloxane Structure

**DOI:** 10.3390/ma13173742

**Published:** 2020-08-24

**Authors:** Xiaozhou Xu, Yi Liu, Bangwei Lan, Song Mo, Lei Zhai, Minhui He, Lin Fan

**Affiliations:** 1 Key Laboratory of Science and Technology on High-Tech Polymer Materials, Institute of Chemistry, Chinese Academy of Sciences, Beijing 100190, China; xuxiaozhou@iccas.ac.cn (X.X.); liuyipi@iccas.ac.cn (Y.L.); lanbangwei@iccas.ac.cn (B.L.); mosong@iccas.ac.cn (S.M.); zhailei@iccas.ac.cn (L.Z.); heminhui@iccas.ac.cn (M.H.); 2 School of Chemical Science, University of Chinese Academy of Sciences, Beijing 100049, China

**Keywords:** phenylethynyl-terminated oligoimide, siloxane structure, isomeric dianhydrides, rheological properties, thermal stability

## Abstract

A series of 4-phenylethnylphthalic anhydride (PEPA)-terminated oligoimides were prepared by co-oligomerizing isomeric dianhydrides, i.e., 2,3,3′,4′-biphenyltetracarboxylic dianhydride (a-BPDA), 2,3,3′,4′-benzophenonetetracarboxylic dianhydride (a-BTDA) or 2,3,3′,4′-diphenylethertetracarboxylic dianhydride (a-ODPA), with diamines mixture of bis(4-aminophenoxy)dimethyl silane (APDS) and 2,2′-bis(trifluoromethyl) benzidine (TFDB). The effects of siloxane content and dianhydride structure on the rheological properties of these oligoimides and thermal stability of the corresponding cured polyimide resins were investigated. The results indicated that the introduction of the siloxane structure improved the melt processability of the oligoimides, while the thermal stability of the cured polyimide resins reduced. The oligoimide derived from a-ODPA revealed better melt processability and melt stability due to the existence of a flexible dianhydride structure. The oligoimide PIS-O10 derived from a-ODPA gave the lowest minimum melt viscosity of 0.09 Pa·s at 333 °C and showed the excellent melt stability at 260 °C for 2 h with the melt viscosity in the range of 0.69–1.63 Pa·s. It is also noted that the thermal stability of these resins can be further enhanced by postcuring at 400–450 °C, which is attributed to the almost complete chemical crosslinking of the phenyethynyl combined with oxidative crosslinking of siloxane. The PIS-T10 and PIS-O10 resins that were based on a-BTDA and a-ODPA, respectively, even showed a glass transition temperature over 550 °C after postcuring at 450 °C for 1 h.

## 1. Introduction

Thermosetting polyimide resins have been successfully used as the matrix resin of composites in the manufacture of aviation and aerospace vehicles, owing to their excellent thermal stability, high mechanical properties and good dielectric properties [1,2,3,4]. However, they are usually difficult to process because of the poor melt fluidity and the volatiles release [5,6]. Therefore, the high temperature and high pressure molding process or vacuum autoclave molding are usually applied for the fabrication of composite parts. Although these fabrication technics are well realized for processing the large and simple parts of polyimide matrix composites, the problems, such as environmental pollution, low production efficiency and high processing cost, still restrict their wide application. Resin transfer molding (RTM) offers an alternative option for the fabrication of polyimide matrix composites, however, requires the resin to possess a sufficient melt fluidity, good melt stability, and no volatile evolution during the resin processing [7]. In past decades, many efforts have been made to improve the melt processability of the thermosetting polyimide resins while attempting to maintain their excellent thermal stability and good mechanical properties.

The oligoimides terminated with 4-phenylethnylphthalic anhydride (PEPA) have been successfully developed since the 1990s, which exhibited a wide processing window, cured without volatiles evolution and provided the polymer with good toughness after curing [8,9,10,11]. More attention has been paid to the PEPA-terminated oligoimides for RTM processing in recent years. In order to afford the oligoimides’ low melt viscosity and good melt stability, many works have been done by incorporating a flexible structure in the backbone of the oligoimide and reducing the molecular weight of oligoimides. However, the flexible main chain structure will sacrifice the thermal resistance of polyimide resins and the low molecular weight design will damage their toughness [12].

Connell and his coworkers reported the PEPA-terminated oligoimides based on isomeric dianhydride 2,3,3′,4′-benzophenonetetracarboxylic dianhydride (a-BPDA) and found that the incorporation of symmetric and nonplanar structures into polyimide backbone could achieve good balance between heat resistance and melt processability [13]. It is considered that the asymmetric structure of a-BPDA decreases the intermolecular forces of the resulting oligoimides. Meanwhile, the rotational energy barrier of cured polyimides is increased during the glass relaxation process [14]. Many kinds of PEPA-terminated oligoimides have been investigated for RTM processing until now. Among these resins, PETI-375, which derived from isomeric dianhydride of a-BPDA as well as diamines of 1,3-bis(4-aminophenoxy)benzene (1,3,4-APB) and TFDB endcapped with PEPA, exhibited an extremely good balance of melt processability and thermal stability with a stable melt viscosity of 0.1–0.4 Pa·s at 280 °C and *T*_g_ as high as 375 °C [15]. Chuang et al. synthesized the PEPA-terminated polyimides with excellent melting performance from 2,3,3′,4′-benzophenonetetracarboxylic dianhydride (a-BTDA) [16]. They suggested that the a-BTDA-based oligoimides exhibited a low melt viscosity of 1–2 Pa·s and revealed high thermal stability with *T*_g_ of 330–400 °C. Moreover, their composites fabricated by RTM process showed an outstanding thermooxidative stability at 288–327 °C.

Additionally, the introduction of a second crosslinkable segment in the main chain of thermosetting polyimide, besides the PEPA crosslinking agent, has also been studied in order to further improve the heat resistance of polyimide resins and maintain simultaneously the melt processability of its oligoimides. Fernberg et al. developed a 6FDA-based phenylethynyl-terminated polyimide resin MHT-R suitable for RTM by introducing ethynyl bis-phthalic anhydride (EBPA) into the main chain [17,18]. They found that MHT-R resin exhibited relatively low melt viscosity of 0.2–2.0 Pa·s between 250–320 °C, which is suitable for fabricating high-quality carbon fiber-reinforced composites by RTM. The *T*_g_ of the resin can reach 460 °C by making the best of crosslinking reaction of the ethynyl group in the PEPA and in the dianhydride EBPA. The high-quality MHT-R matrix composites could withstand up towards 350–400 °C. However, there are few other crosslinking reactions are used to develop the polyimide resin suitable for RTM with higher thermal stability.

In our previous works on the PEPA-terminated polyimides, we found that the melt processability of oligoimides could be improved by the introduction of siloxane structure; meanwhile, the thermal stability of resultant polyimide was not only determined by the phenyethynyl crosslinking but also affected by the oxidative crosslinking of siloxane [19]. As an effort to develop the thermosetting polyimide resins for RTM process with excellent comprehensive of processability and thermal properties, in this paper, a series of PEPA-terminated oligoimides with different siloxane content and different isomeric dianhydrides were designed and synthesized. The isomeric dianhydrides of a-BPDA, a-BTDA or a-ODPA, the fluorinated diamine of TFDB and the siloxane diamine of APDS are incorporated to afford the oligoimides excellent melt processability. In addition, it is expected that APDS could provide the polyimide resins with improved thermal stability through high-temperature oxidative crosslinking. The effect of siloxane content and dianhydride structure on the processability of the oligoimides, the mechanical and thermal properties of the cured polyimide resins were investigated. The dependence of their thermal properties on the postcuring conditions and the correlation with the structure evolution of siloxane were also discussed.

## 2. Materials and Methods

### 2.1. Materials

4-Phenylethynylphthalic anhydride (PEPA) and 2,2′-bis(trifluoromethyl)benzidine (TFDB) were purchased from Changzhou Sunlight Fine Chemicals Co., Ltd., Changzhou, China, and dried under vacuum for 12 h at 120 °C prior to use. 2,3,3′,4′-biphenyltetracarboxylic dianhydride (a-BPDA, Chenxing Chemical, Yancheng, China), 2,3,3′,4′-benzophenonetetracarboxylic dianhydride (a-BTDA, Bohai Chemical, Wuxi, China) and 2,3,3′,4′-diphenylethertetracarboxylic dianhydride (a-ODPA, Shanghai Research Institute of Synthetic Resins, Shanghai, China) were obtained from commercial suppliers and dried under vacuum for 12 h at 120 °C prior to use. Bis(4-aminophenoxy)dimethyl silane (APDS) was purchased from Gelest (A Meryer Chemical Technology Shanghai Company, Shanghai, China) and used as received. Commercially available N-methylpyrrolidone (NMP) was purified by vacuum distillation prior to use. Toluene was used as received.

### 2.2. Synthesis of PEPA-Terminated Oligoimides Containing Siloxane Structure

**Scheme 1:**
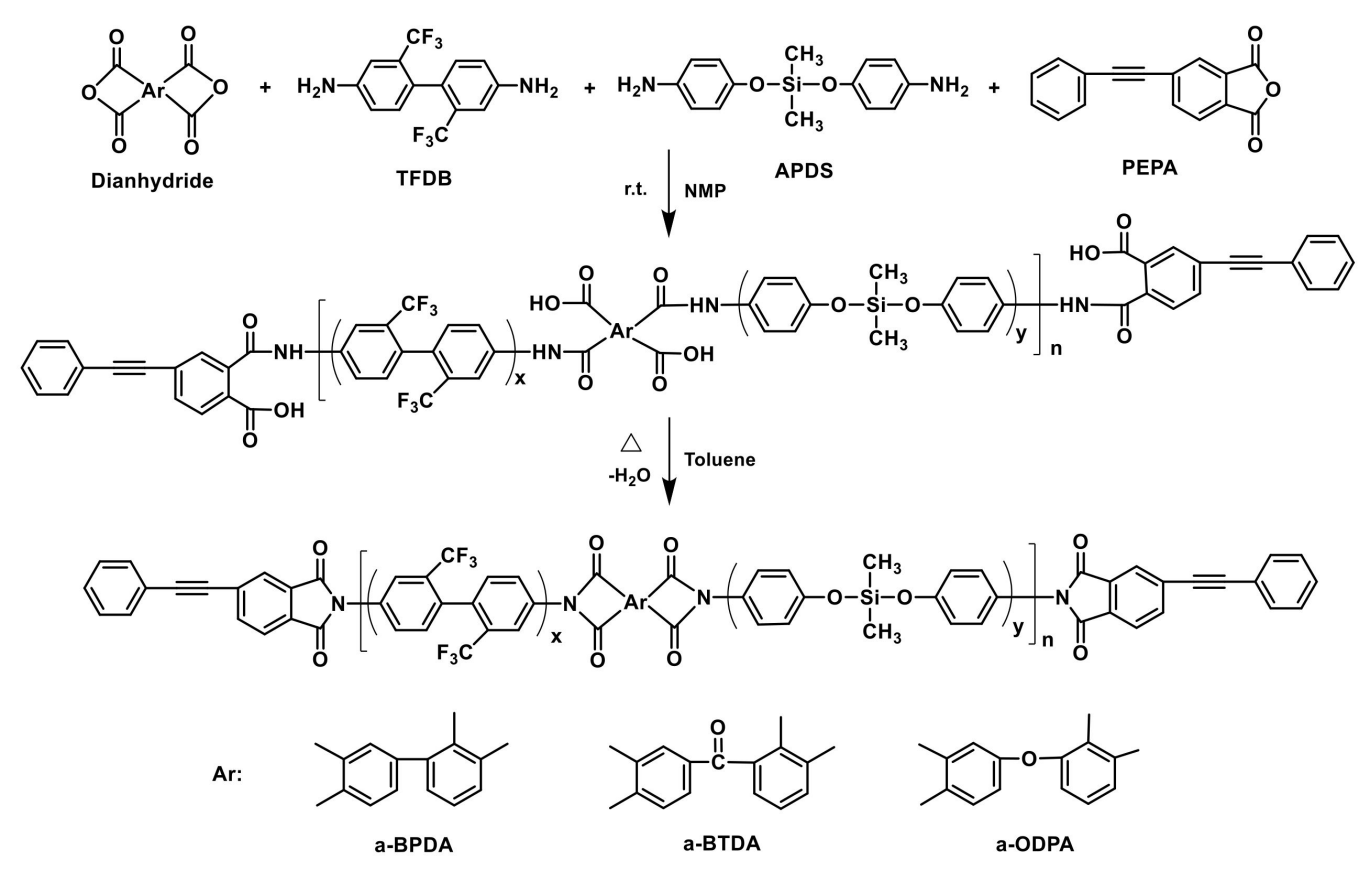
Synthesis of PEPA-terminated oligoimides containing siloxane structure.

A series of PEPA-terminated oligoimides with siloxane structure from diamines of TFDB and APDS as well as various dianhydrides, i.e., a-BPDA, a-BTDA and a-ODPA, were synthesized (Scheme 1). The oligoimides derived from a-BPDA with APDS molar ratio of 0%, 10%, 20% and 30% in diamine were defined as PIS-A0, PIS-A10, PIS-A20 and PIS-A30, respectively. The oligoimides based on a-BTDA and a-ODPA with APDS molar ratio of 10% in diamine were defined as PIS-T10 and PIS-O10, respectively. In a typical experiment, a three-necked flask equipped with a mechanical stirrer, water condenser, Dean–Stark trap and nitrogen inlet/outlet was placed TFDB (17.29 g, 54.0 mmol) and APDS (1.65 g, 6.0 mmol) as well as 50 g of NMP. The mixture was stirred until the diamines completely dissolved to produce a homogeneous solution. Then, the slurry of a-BPDA (8.83 g, 30.0 mmol) and PEPA (14.89 g, 60.0 mmol) in 25 g of NMP was added. The reaction mixture was adjusted to 30 wt% of solid content by adding 24.5 g of NMP and stirred in nitrogen at room temperature for 12 h. After that, 10 g of toluene was added into the reaction solution and the mixture was heated and refluxed at 180 °C for 8–12 h and, in the meantime, the water evolved in the thermal imidization was removed by azeotropic distillation. After cooling down to 100–120 °C, the reaction solution was poured into excess ethanol to produce white precipitate, which was then collected by filtration, washed repeatedly with ethanol, and dried in vacuum at 220 °C for 4 h. The grey yellow solid powder of PIS-A10 oligoimide was obtained (yield: 39.29 g, 97%).

A series of PIS-A oligoimides from a-BPDA with 0, 10, 20 and 30% of APDS molar ratio in diamines and the oligoimides derived from different dianhydrides, such as a-BTDA and a-ODPA, were synthesized in the similar procedure as described above, respectively. The designed degree of polymerization for these oligoimides was 1.0 and their theoretical calculated average molecular weights were in the range of 1344–1399 g/mol. The nomination, chemical compositions and theoretical molecular weight of these oligoimides were listed in Table 1.

### 2.3. Preparation of Cured Polyimides

The oligoimide powders were put in a stainless steel mold, which was then placed in a hot press preheated at 250 °C. The mold was heated to 300 °C at a ramp rate of 4 °C/min and kept there for 20 min. Then, the mold was heated to 360 °C at a ramp rate of 3 °C/min and kept for 10–30 min, and a pressure of 1.5 MPa was applied gradually. After that, the mold was heated to 370 °C and kept for 2 h. After the mold was cooled to 200 °C, the cured polyimide sheet with the dimension of 80 mm × 80 mm × 2 mm was removed from the mold. In the postcuring procedure, the cured polyimide sheets were thermally treated in the air oven at the corresponding temperature and time.

### 2.4. Characterization and Measurements

The number-average molecular weights (*M*_n_) and the polydispersities (*M*_w_/*M*_n_) of these oligoimides were measured by a gel permeation chromatography (GPC) system equipped with a model 1515 HPLC pump (Waters, Milford, MA, USA), three Waters Styragel columns (HT-2 × 2 and HT-4) and a Waters 2414 refractive index detector. NMP with 0.02 M LiBr was used as eluent at a flow rate of 0.8 mL/min. The sample concentration was 5 mg/mL and monodisperse polystyrene standards were used for the calibration. The chemical structure of oligoimides were evaluated by Fourier transform infrared (FT-IR) on a Perkin-Elmer 782 Fourier transform infrared spectrometer (Bruker, Hong Kong, China) with pressed KBr pellets. X-ray diffraction (XRD) patterns were detected by a Ragaku D/MAX-2500 X-ray powder diffraction (PANalytical B.V., Almelo, The Netherlands) with Cu-Kα incident radiation (λ = 1.5418 Å). Rheological measurements were tested by an AR2000 rheometer of TA Instrument (New Castle, DE, USA). The specimen discs with a diameter of 25 mm and a thickness of 1.3–1.5 mm were prepared by press molding the oligoimide powder. In the measurement, the specimen discs were loaded in the parallel plates, in which the top parallel plate was oscillated at a fixed angular frequency of 5 rad/s and a fixed strain of 0.1%. To study the dynamic rheological behavior of the oligoimides, the specimen discs were scanned from 200 to 400 °C at a rate of 4 °C/min. The isothermal viscosity of the oligoimides were determined measured at the 260 °C for 2 h. Differential scanning calorimetry (DSC) (New Castle, DE, USA) and dynamic mechanical analysis (DMA) (New Castle, DE, USA) in nitrogen atmosphere were carried out by TA Q100 at a rate of 10 °C/min and TA Q800 at a rate of 5 °C/min, respectively. Thermogravimetric analysis (TGA) (New Castle, DE, USA) in the air was performed on a TA Q50 (New Castle, DE, USA) a rate of 20 °C/min atmosphere. Instron 5567 tensile tester (Norwood, MA, USA) was used to assess the mechanical properties of the cured polyimides. The tensile and flexural properties were carried out in accordance with GB/T 1040.2-2006 and GB/T 9341-2008, respectively. The X-ray photoelectron spectroscopy (XPS) (VG Instruments, Altrincham, Cheshire, England) was determined by an ESCALab 220i-XL with an Al Kα excitation source. Change correction was carried out by the C 1s core line setting the hydrocarbon signal to 284.6 eV.

## 3. Results and Discussion

### 3.1. Characterization of PEPA-Terminated Oligoimides Containing Siloxane Structure

**Figure 1:**
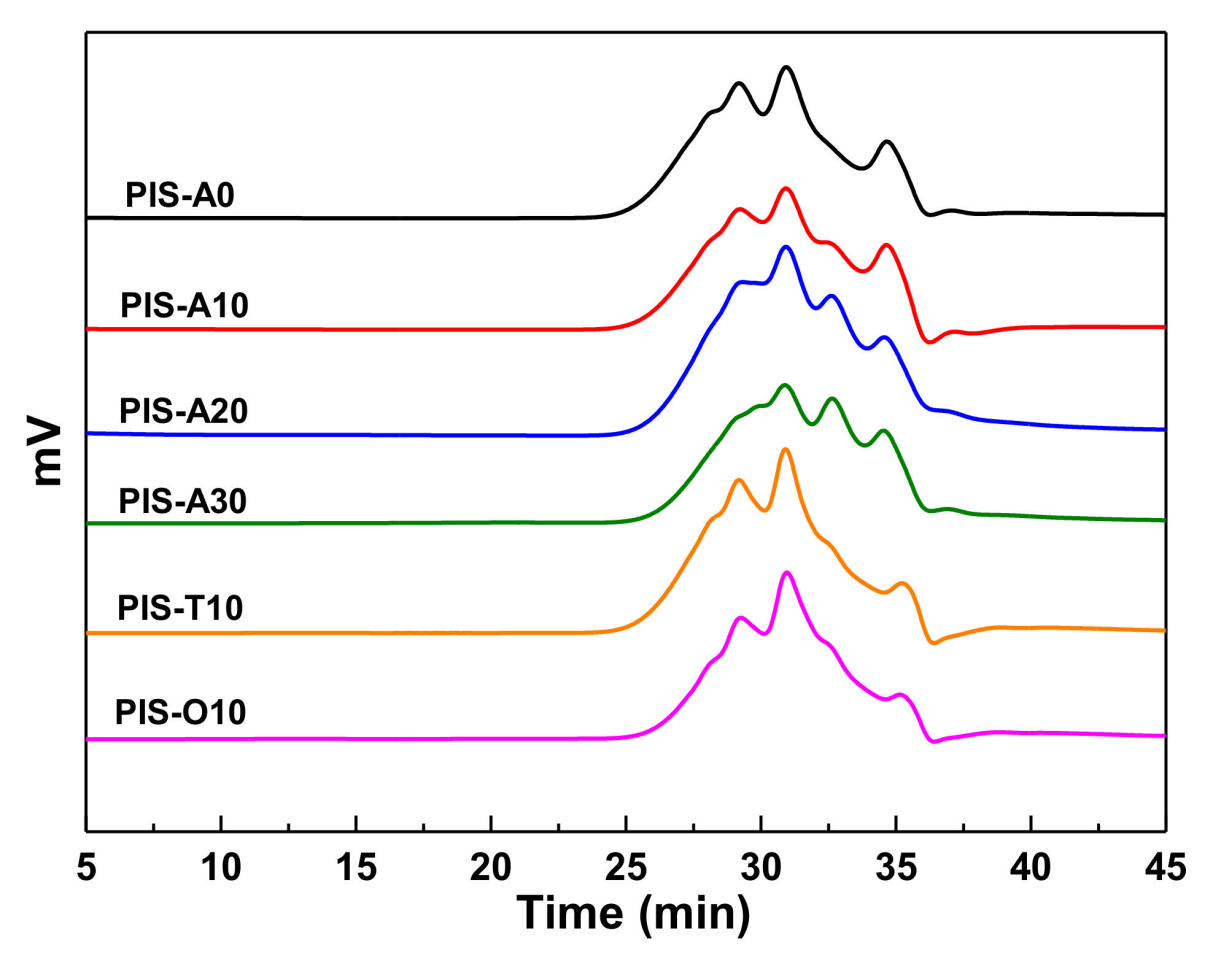
GPC curves of PEPA-terminated oligoimides containing siloxane structure.

The GPC analyses were performed on the oligoimides to assess the *M*_n_ and the polydispersities (*M*_w_/*M*_n_), as shown in Figure 1 and the corresponding data were summarized in Table 1. The GPC curves showed that each oligoimide consisted of several elution time peaks between 25–37 min, implying the composition of several chemical species. The *M*_n_ values measured by GPC were slightly greater than the theroretical ones because of the polarity difference between measured samples and polystyrene standards [20]. The polydispersities of these oligoimides were in the range of 1.43–1.51. It is also found that PIS-A10, PIS-T10 and PIS-O10 mainly consist of three elution time peaks at 29, 31 and 35 min, respectively. However, these elution time peaks were detected at relatively longer elution time for the oligoimides with higher siloxane content, suggesting the existence of more low-molecular-weight species. It is interesting to note that the measured *M*_n_ of PIS-A oligoimides decreased with the increasing of siloxane content. For instance, PIS-A0 without APDS exhibited the measured *M*_n_ of 1687 g/mol, while the PIS-A30 with 30% of APDS molar fraction in diamines exhibited the measured *M*_n_ of 1356 g/mol. The number-average molecular weight difference Δ*M*_n_ between the measured *M*_n_ and the calculated one gradually decreased with the increasing siloxane content in the order of PIS-A0 (323 g/mol) > PIS-A10 (172 g/mol) > PIS-A20 (79 g/mol) > PIS-A30 (12 g/mol). This is probably because of the relatively lower nucleophilic reactivity of APDS, which leads to the oligoimides with lower measured *M*_n_.

**Figure 2:**
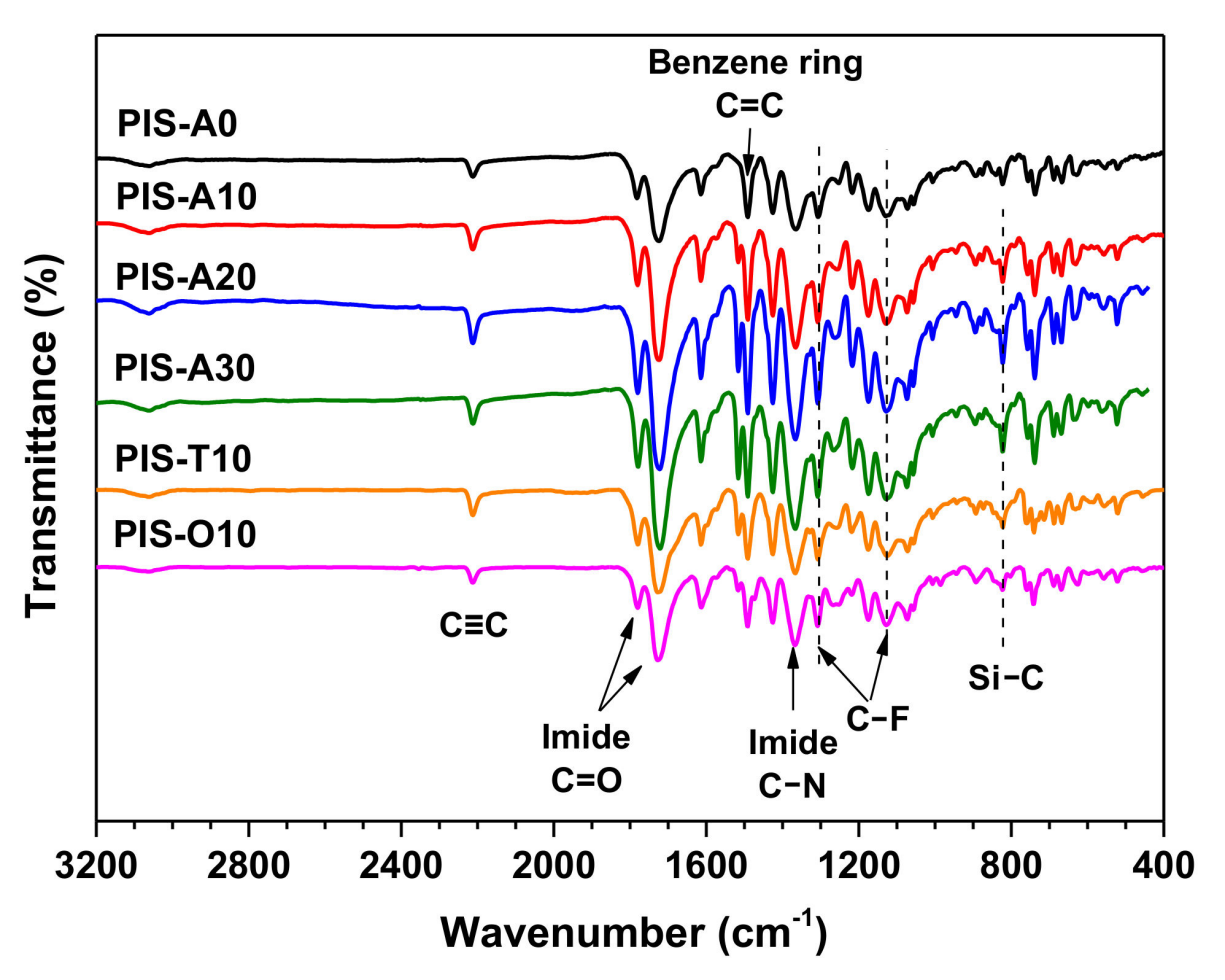
FT-IR spectra of PEPA-terminated oligoimides containing siloxane structure.

The chemical structure of the PEPA-terminated oligoimides containing siloxane structure were detected by FT-IR. As shown in the Figure 2, all the oligoimides had similar absorption peaks. The strong absorption peak near 2210 cm^−1^ was associated with the C≡C stretching vibration of phenylethynyl group. The absorption peaks near 1780 and 1720 cm^−1^ were attributed to asymmetric and symmetric stretching vibration peaks of C=O on the imide ring, respectively [21]. The characteristic absorption of 1370 cm^−1^ was related to the stretching vibration of imide C–N. Moreover, the absorption at 1500 cm^−1^ of C=C due to benzene ring as well as the absorption at 1303 and 1120 cm^−1^ of C–F stretching vibration were also observed [22]. However, the absorption at 1100 cm^−1^ of Si–O band was difficult to detect because of the overlap of C–F band at 1120 cm^−1^ and only the weak absorption around 820 cm^–1^ attributed to the stretching vibration of Si–C was observed for these oligoimides except PIS-A0 [23,24]. The Si–C absorption was shown to be slightly stronger with the increase of siloxane content. From the analysis of GPC and FT-IR, it can be inferred that the oligoimides have the designed number-average molecular weights and chemical structures.

**Figure 3:**
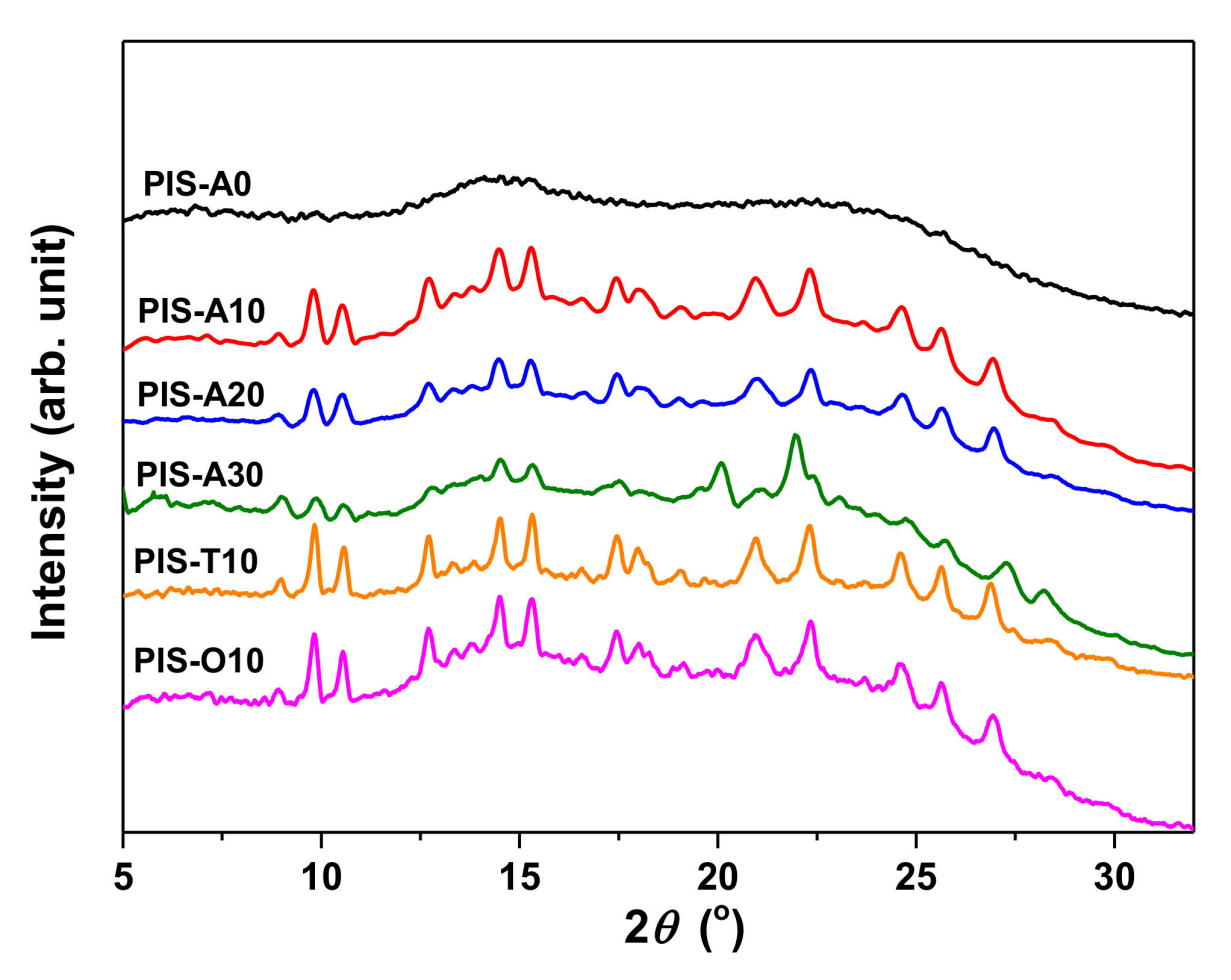
XRD patterns of PEPA-terminated oligoimides containing siloxane structure.

The XRD patterns of the oligoimides were shown in Figure 3. It can be seen that these oligoimide containing siloxane structure showed some crystalline peaks between 2*θ* angle 8°–28° except PIS-A0. This may be related to the low molecular weight of these oligoimides combined with the existence of flexible siloxane, which made the molecular chain easier moving and packing to form the ordered crystalline.

### 3.2. Processability of PEPA-Terminated Oligoimides Containing Siloxane Structure

**Figure 4:**
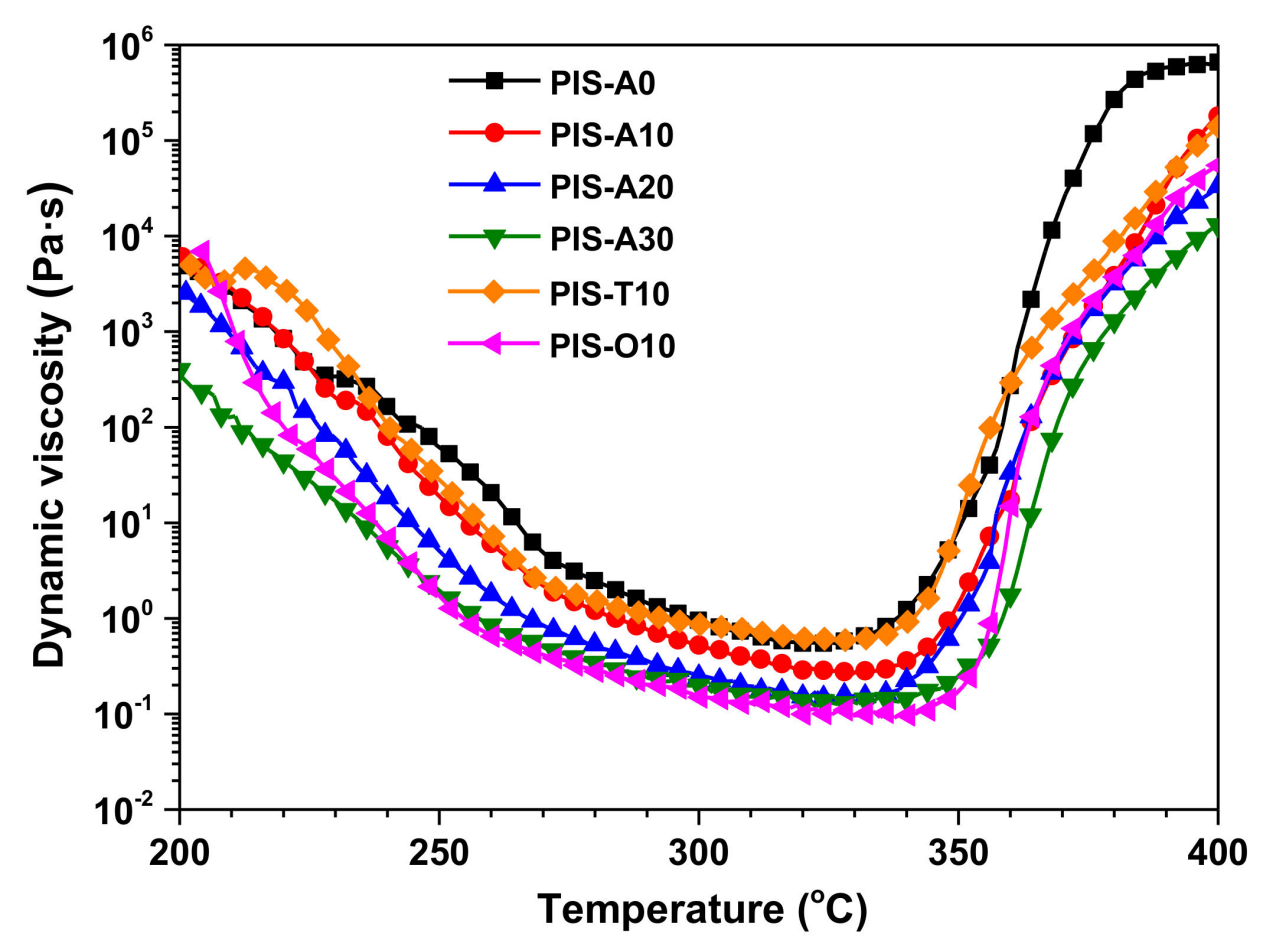
Dynamic viscosity of PEPA-terminated oligoimides containing siloxane structure.

Resin transfer molding (RTM) put forward severe requirement for resin processing performance. Therefore, the key factors affecting the preparation of composites by RTM are the lower melt viscosities and excellent melt stability of oligoimides. The dynamic rheological behavior of the oligoimides are shown in Figure 4, and the corresponding dynamic viscosity data were listed in Table 2. The dynamic viscosity of all oligoimides dropped abruptly with the temperature increasing from 200 to 260 °C due to the softening of backbone, which then decreased gradually until the temperature enhanced to 330 °C. As the scanning temperature was over 350 °C, the dynamic viscosity increased abruptly due to the crosslinking of phenylethynyl group. Moreover, it can be seen that PIS-A oligoimides showed the lower minimum melt viscosity with the increase of siloxane content, which is 0.55 Pa·s for PIS-A0 and 0.12 Pa·s for PIA-A30, respectively. Comparing the oligoimides with different dianhydride structure, it is found that their minimum melt viscosity dropped in the order of PIS-T10 > PIS-A10 > PIS-O10. The PIS-O10 oligoimide derived from a-ODPA containing flexible ether linkage gave the minimum melt viscosity as low as 0.09 Pa·s at 333 °C. It is worth to mention that both of PIS-A30 and PIS-O10 oligoimides revealed the melt viscosity no more than 1 Pa·s in the temperature range of 260–280 °C, implying the broader processing window.

**Figure 5:**
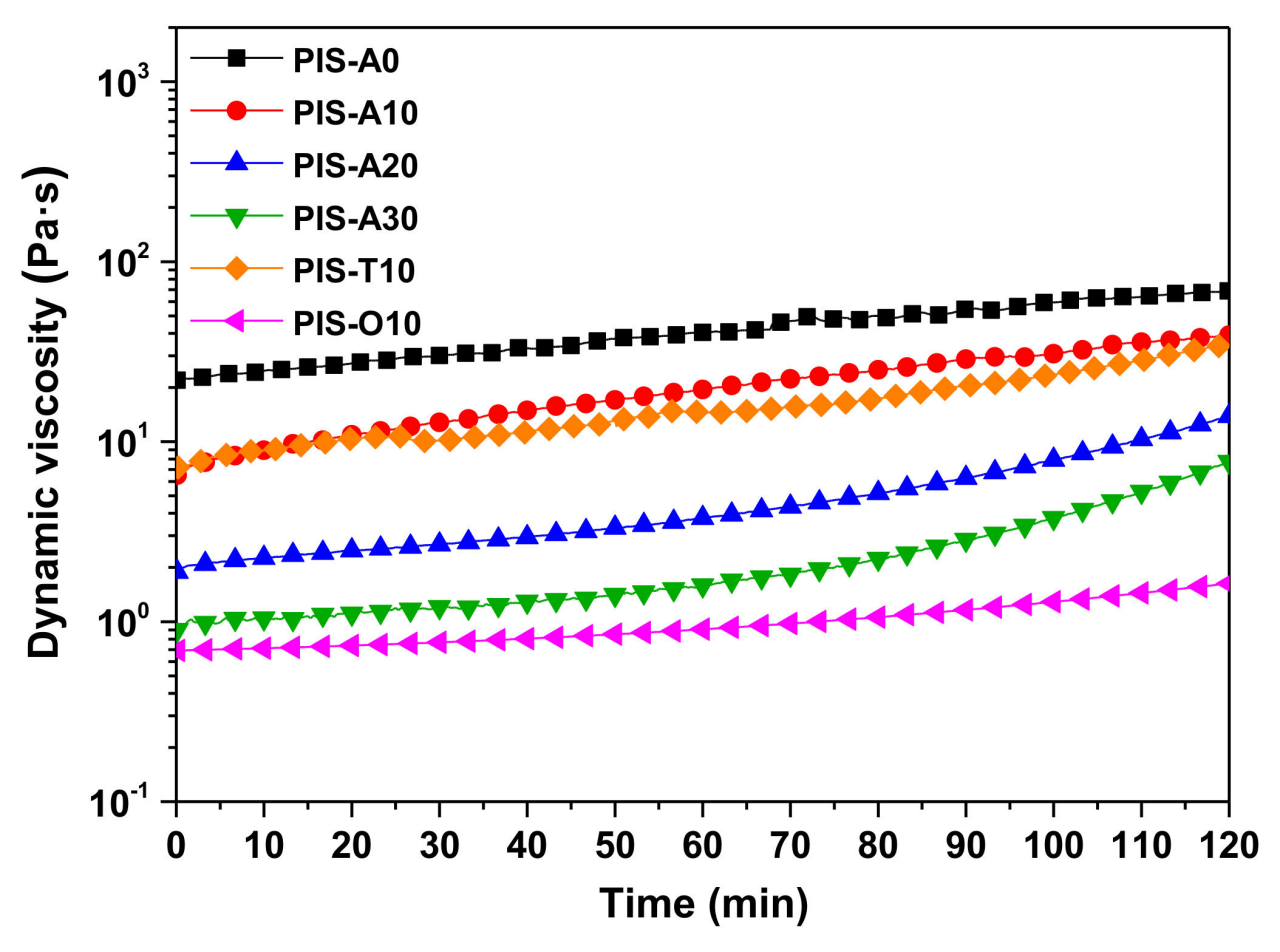
Isothermal viscosity behavior of PEPA-terminated oligoimides containing siloxane structure at 260 °C for 2 h.

The melt stability of oligoimides has a great influence on the processing of the fabrication of composites. In order to investigate the melt stability of the oligoimides, the isothermal melt viscosity measurements at 260 °C were conducted. Figure 5 shows the dynamic viscosity curves of these oligoimides as a function of time, and the corresponding melt viscosity variations are summarized in Table 2. It can be seen that the melt viscosity of all the oligoimides increased with the isothermal measurement at 260 °C for 2 h. The PIS-A0 oligoimide without siloxane structure revealed the largest melt viscosity variation of 21.92–69.09 Pa·s, while PIS-A30 with relatively higher siloxane content gave the better melt stability with the melt viscosity variation of 0.90–7.74 Pa·s. In addition, the order of contribution of dianhydride structure to melt stability of the oligoimides was a-ODPA > a-BTDA > a-BPDA. The PIS-O10 had the most excellent melt stability with the slightly change of melt viscosity from 0.69 to 1.63 Pa·s during the isothermal treatment at 260 °C for 2 h. We suspected that the fractional free volume of the oligoimides maybe affect their melt stability.

**Figure 6:**
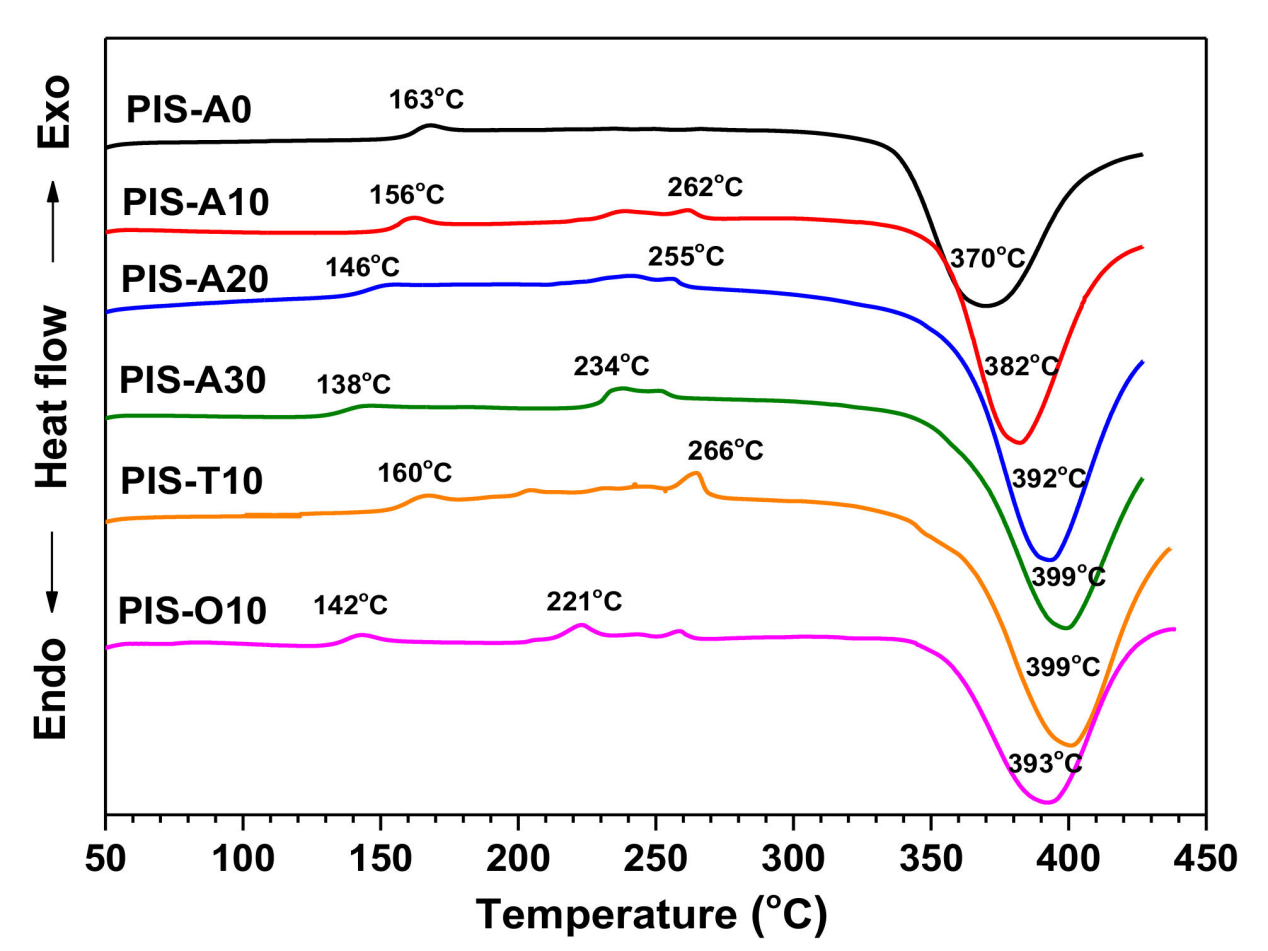
DSC curves of PEPA-terminated oligoimides containing siloxane structure.

Figure 6 compared the DSC curves of oligoimides with different siloxane content and different isomeric dianhydrides. It is noted that these oligoimides revealed a weak *T*_g_ in the range of 138–163 °C. Moreover, these oligoimides with higher siloxane content gave the relatively lower *T*_g_. For PIS-A oligoimides, the *T*_g_ of PIS-A30 is 25 °C lower than that of PIS-A0 owing to the existence of flexible siloxane. Comparing the *T*_g_s between the oligoimides with different dianhydride structure, it is found that the PIS-T10 oligoimide gave the higher *T*_g_ value of 160 °C. On the other hand, the PIS-O10 oligoimide showed the lower *T*_g_ value of 142 °C because of the introduction of flexible ether linkage as compared to that of PIS-A10. The additional crystallization melting endothermic peaks in the temperature range of 220–270 °C were also detected for these oligoimides, except for PIS-A0. Combined with the crystal diffraction peaks observed on the XRD patterns, it can be concluded that the endothermic peaks are associated with the melting of crystallites [25,26]. These oligoimides also exhibited the exothermic peaks at 370–399 °C, which can be attributed to the crosslinking reaction of the phenylethynyl group. Moreover, it is also noticed that the exothermic peaks of PIS-A oligoimides were detected at the higher temperature accompanied with the siloxane content increasing. It is suspected to be related to the ether structure, which has some degree retarding impacts on the curing reaction of phenylethynyl group [27]. The PIS-A30 with the highest ether bond loading showed the highest exothermic peak value at 399 °C.

### 3.3. Mechanical and Thermal Properties of the Cured Polyimides

These PEPA-terminated oligoimides were molded using the hot press at 370 °C for 2 h to gain the cured polyimide sheet. Table 3 summarized the mechanical properties of the cured polyimides at room temperature. High-quality polyimide sheets can be obtained except PIS-A30 because it is not tough enough to fabricate the testing specimen. This may be related to the relatively low molecular weight of PIS-A30 oligoimide, resulting in the brittleness of cured polyimide resin [28,29]. Brostow et al. formulated a quantitative definition of brittleness in terms of elongation at break [30,31]. They think there is an inverse relationship between the brittleness and elongation at break. Our results are also consistent with their research. The cured polyimides prepared from these oligoimides with higher siloxane content exhibited a slight decrease in toughness because of the relatively lower molecular weight and higher crosslinking density. For example, PIS-A0 exhibited good mechanical properties with the tensile strength of 70.48 MPa, flexural strength of 121.52 MPa, and elongation at breakage of 3.90%, while those for PIS-A20 decreased to 62.28 MPa, 99.37 MPa and 2.77%, respectively. Comparing the cured polyimides based on different isomeric dianhydrides with same siloxane content, it is found that they exhibited the moderate tensile and flexural strengths in the ranges of 54.69–62.82 and 102.78–114.07 MPa, respectively. The cured PIS-T10 gave the highest tensile and flexural modulus because of the rigidity of dianhydride.

**Figure 7:**
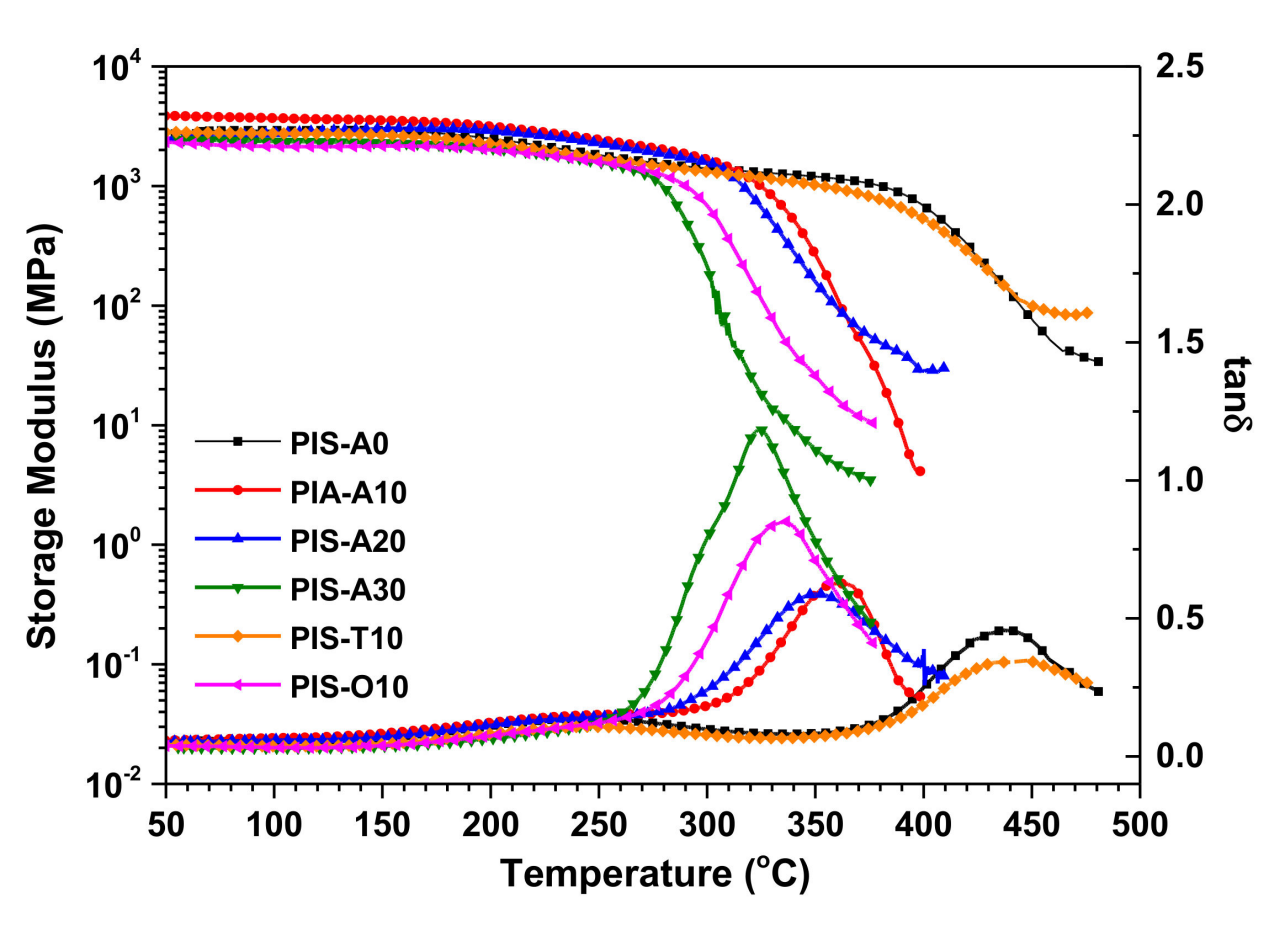
DMA curves of the cured polyimide resins.

The thermal properties of these cured polyimide resins were detected through DMA and TGA. The DMA curves of these cured polyimide resins are shown in Figure 7, in which the storage modulus curves firstly decreased gradually and then dropped rapidly with the temperature increasing. In the tan*δ* curves, the weak subglass relaxation peaks owing to 2,2′-disubstitutes of benzidine moiety with -CF_3_ were observed around 250 °C [32], meanwhile, the sharp glass relaxation peaks related to the movement of polyimide backbone were also detected at 323–442 °C. It is noticed that these cured polyimide resins exhibited an obvious decrease in *T*_g_s with the increase of siloxane content. From the DMA data summarized in Table 4, it is found that the difference in *T*_g_ between PIS-A0 and PIS-A30 is as high as 119 °C. The cured PIS-T10 resin derived from a-BTDA showed the relatively higher *T*_g_ of 437 °C as compared with the cured PIS-A10 and PIS-O10 resins with similar siloxane content. Chuang et al. reported that the planar sp^2^ bonding *in* carbonyl group (C=O) is more rigid than the sp^3^ tetrahedral bonding linkage [33]. They also suggested that the rotational barrier of a-BTDA may be higher than that of a-BPDA because carbonyl group in a-BTDA imparts greater rigidity to the polymer backbone [16]. Therefore, the good thermal stability of cured PIS-T10 may be owing to the carbonyl group in the isomeric dianhydride, which increases the energy for the crankshaft motion of cured polyimide resin and provides the high thermal stability as a result.

**Figure 8:**
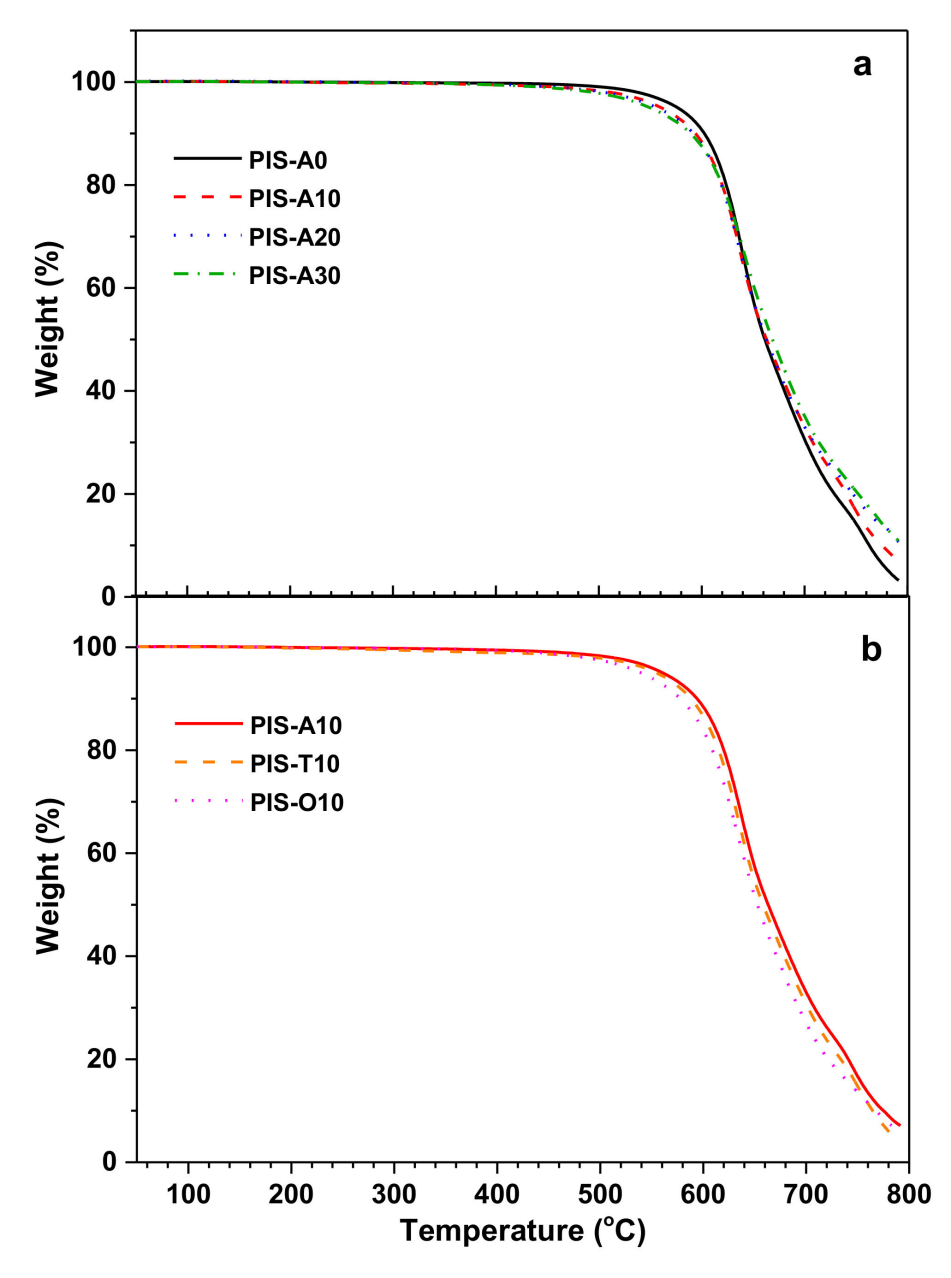
TGA curves of the cured polyimide resins with different siloxane content (**a**) and different dianhydride structure (**b**) measured in air.

The thermal oxidative stability of these cured polyimide resins was evaluated by TGA measurement in air atmosphere and illustrated in Figure 8. These cured polyimide resins exhibited excellent thermal oxidative stability. They showed no obvious weight loss before 450.0 °C and exhibited the temperature at 5% and 10% loss of the original weight (*T*_5_ and *T*_10_) exceeding 539.7 °C and 576.7 °C, respectively. For cured PIS-A resins, there is a gradual decline in *T*_5_ and *T*_10_ values according with the siloxane content increase. For instance, PIS-A0 revealed the *T*_5_ and *T*_10_ values of 576.7 and 602.5 °C, respectively, while PIS-A30 gave the lower *T*_5_ and *T*_10_ values of 549.2 and 589.4 °C, respectively. This may be associated with the early thermoxidative degradation of Si–C bond in siloxane moiety due to its lower bond dissociation energy than the others [34]. However, it is noteworthy that the cured polyimide resins exhibited a higher char residue at 700 °C along with the increasing siloxane content. Their unexpected thermal degradation behavior is probably related to the siloxane oxidation, which leads to a higher char residue. When comparing the TGA results of cured polyimide resins with different isomeric dianhydrides, it is found that *T*_5_ and *T*_10_ values for these resins decreased in the order of PIS-A10 > PIS-T10 > PIS-O10. It is known that the bond dissociation energy of Ar–Ar (450–460 kJ/mol) is greater than that of Ar–CO–Ar (396 kJ/mol) and Ar–O–Ar (330 kJ/mol). As a result, the PIS-A10 displayed relatively higher thermal oxidative stability than the others [35,36].

### 3.4. The Effect of Postcuring Conditions on the Thermal Properties of Cured Polyimides

**Figure 9:**
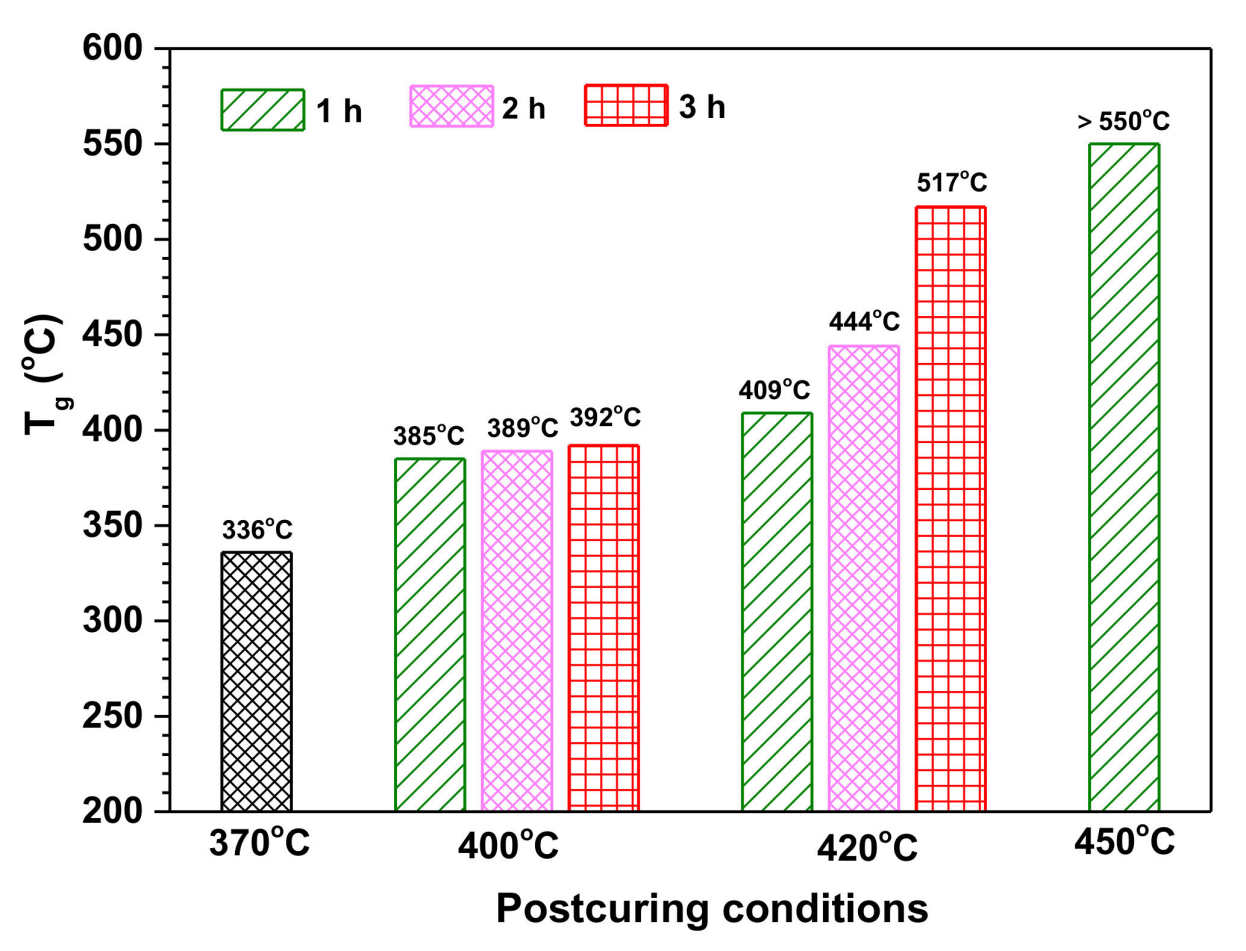
The dependence of *T*_g_ for PIS-O10 on postcuring temperature and time.

In our previous research, it has been confirmed that the thermal stability of polyimide resins containing siloxane structure can be improved by postcuring at a high temperature [37]. Taking the cured PIS-O10 resin as an example, it was postcured in an air atmosphere at 400, 420, and 450 °C for different times, respectively. Its thermal properties and structural evolution of siloxane before and after postcuring were investigated. The *T*_g_ values of PIS-O10 as a function of postcuring conditions are depicted in Figure 9 and compared with that of PIS-O10 as cured at 370 °C. It is found that the *T*_g_ values enhanced from 336 to 385 °C for the PIS-O10 before and after postcuring at 400 °C for 1 h, which showed only slight increase with the time extension. However, as the postcuring temperature enhanced to 420 °C, PIS-O10 resin showed a significant increase in *T*_g_ values along with the postcuring time. The PIS-O10 resin after postcuring at 420 °C for 3 h gave the *T*_g_ value as high as 517 °C, which is 181 °C higher than that of as cured one. It is unexpected that the glass transition peak of tan*δ* even exceeding the testing temperature range of 550 °C after postcuring at 450 °C. The improved thermal stability of PIS-O10 is supposed to relate to the oxidative crosslinking of siloxane.

**Figure 10:**
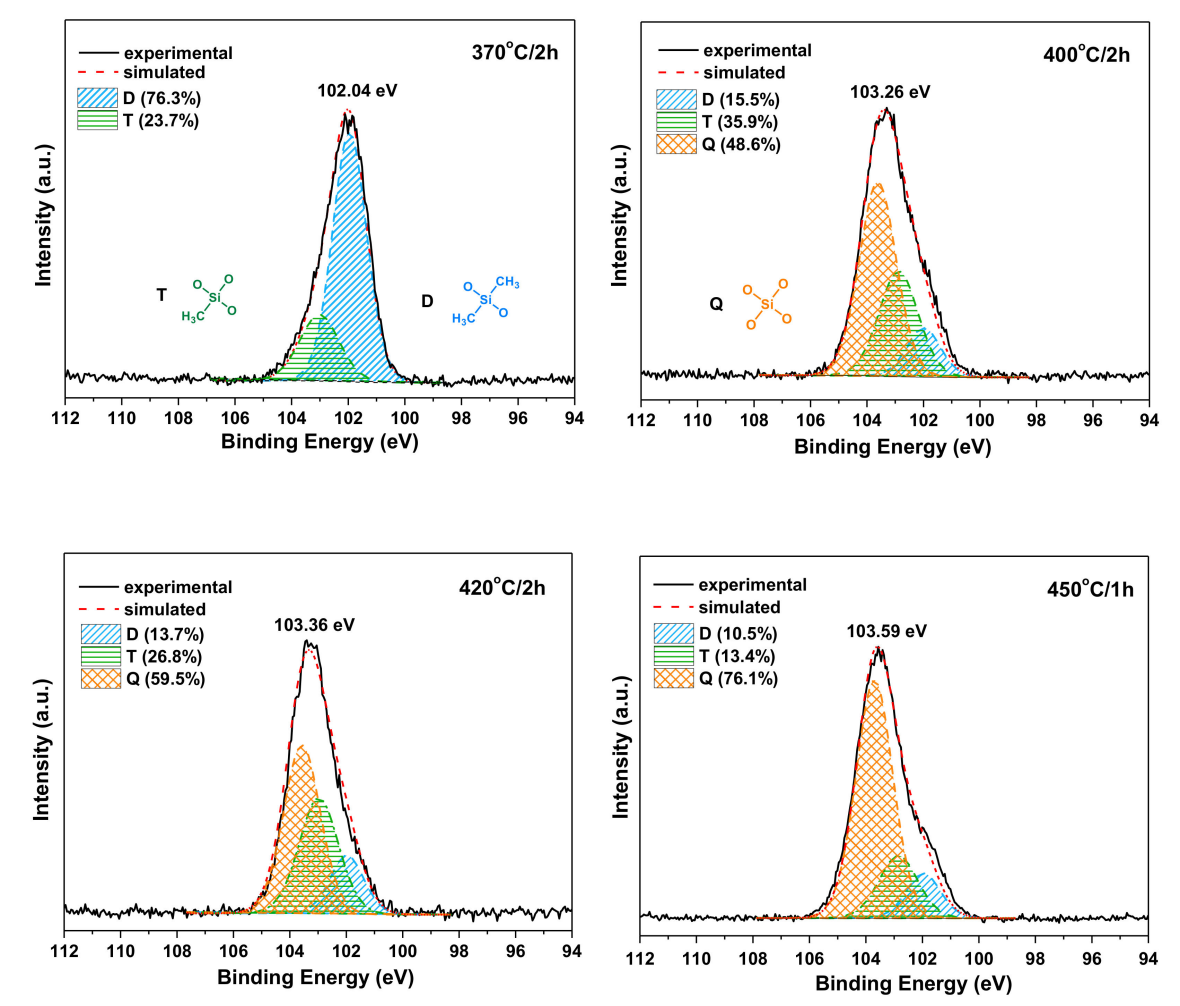
Si 2p XPS spectra of PIS-O10 after curing at 370 °C and postcuring at different conditions.

The structure evolution of siloxane before and after postcuring at different temperature and time was investigated by XPS analysis. As shown in Figure 10, the Si 2p peak was centered at 102.04 eV for PIS-O10 as cured at 370 °C for 2 h, which shifted to the higher binding energy with the increase of postcuring temperature. It is noted that the binding energy of Si 2p for PIS-O10 after postcuring at 450 °C was centered at 103.59 eV. It is well known that when an oxygen atom substitutes an alkyl group attached to the silicon atom, the binding energy of a siloxy unit will increase. Generally, the Si 2p spectra can be fitted with three components, which is the 101.90, 102.90 and 103.50 eV corresponding to the siloxy units of D[(CH_3_)_2_SiO_2/2_], T[CH_3_SiO_3/2_] and Q[SiO_4/2_], respectively [19]. The curve fitting results of the Si 2p peak for as cured PIS-O10 at 370 °C only gave the D and T siloxy units with the concentration of 23.7 and 76.3 at.%, respectively. However, after PIS-O10 was postcured at 400 °C, the Q siloxy units appeared because of the further siloxane oxidation. In the meantime, the increasing intensity of T siloxy units was combined with decreasing D siloxy units. There is more Q component combined with less D and T siloxy units with the increasing of postcuring temperature and extension of postcuring time for PIS-O10. The curve fitting results of Si 2p XPS listed in Table 5 show that PIS-O10 after postcuring at 450 °C for 1h gave the Q siloxy units with a concentration as high as 76.1 at.%, and that for D and T siloxy units, only 10.5 and 13.4 at.%, respectively. The results indicate that the siloxane structure in polyimides undergoes the oxidative crosslinking with the increase of postcuring temperature and time. After PIS-O10 was postcured at 450 °C, most of the siloxane structure is finally converted into inorganic silica structure, which leads to the enhancement of *T*_g_. This can also well explain why polyimide resins with more siloxane content have higher amount of char residue as tested in the air.

**Figure 11:**
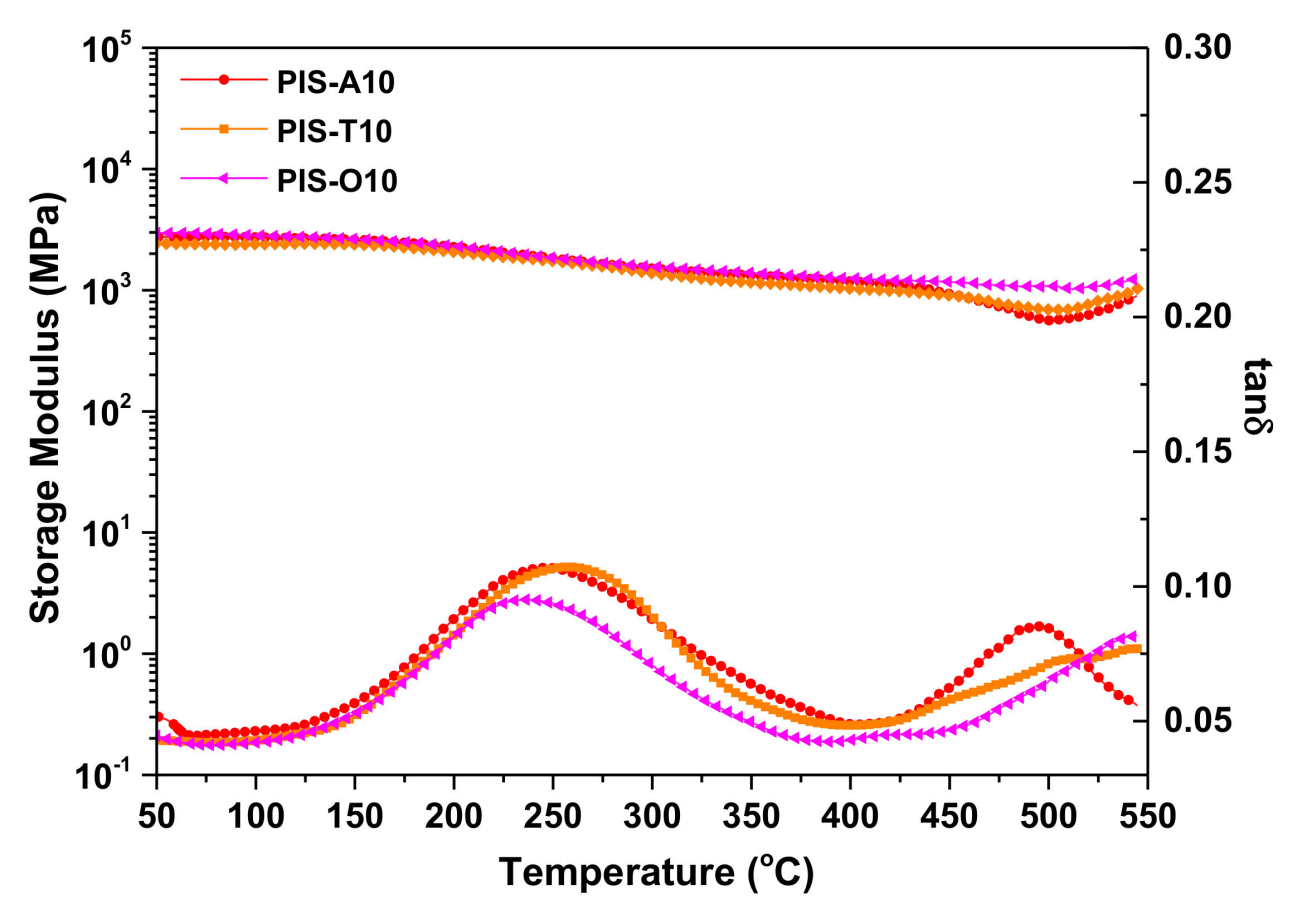
DMA curves of the polyimide resins with different dianhydride structure after postcuring at 450 °C for 1 h.

The inorganic silica-like domains formed after postcuring at elevated temperature can enhance the thermal stability of the polyimide resins with siloxane structure. Therefore, the postcuring effect on the thermal properties of siloxane-containing polyimide derived from different isomeric dianhydrides were investigated. From DMA curves of these polyimide resins after postcuring at 450 °C, shown in Figure 11, it is found that the storage modulus revealed no obvious decrease even as the scanning temperature exceeding 460 °C, suggesting their thermal stability extremely improved. In the tan*δ* curves, the strong subglass relaxation peaks owing to 2,2′-disubstitutes of benzidine moiety with –CF_3_ around 250 °C combined with broad glass transition peaks over 498 °C were detected. Moreover, it is noticed that the glass transition peak of tanδ even exceeding the testing temperature range of 550 °C for PIS-T10 and PIS-O10 after postcuring at 450 °C.

The significant enhancement of thermal stability for these polyimide resins is associated with the deep crosslinking of phenylethynyl group and further oxidative crosslinking of siloxane. The corresponding TGA data of polyimide resins with different isomeric dianhydrides after postcuring at 450 °C were listed in Table 6. These postcured resins exhibited better thermal oxidative stability with the *T*_5_ > 573.4 °C and *T*_10_ > 595.1 °C, which are 16.4–33.2 and 5.2–17.3 °C higher than those for as cured ones.

**Figure 12:**
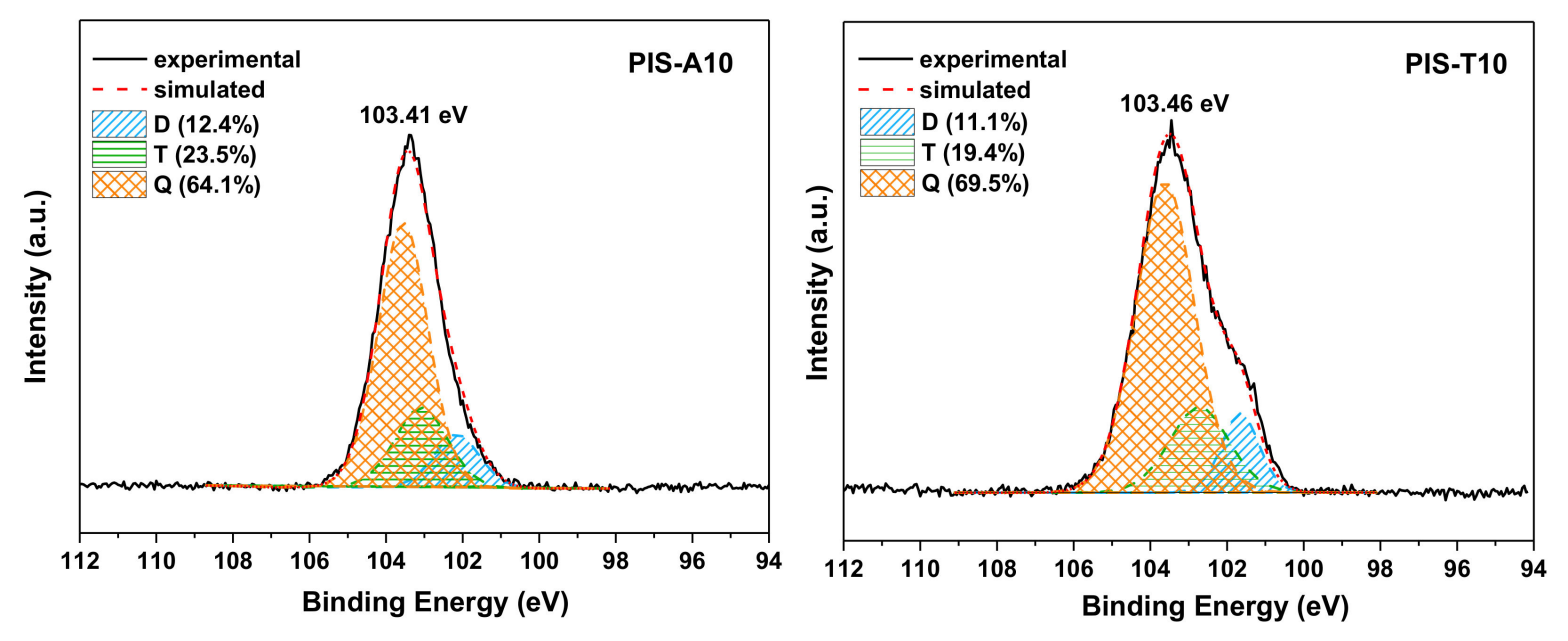
Si 2p XPS spectra of PIS-A10 and PIS-T10 resins after postcuring at 450 °C for 1 h.

The oxidative crosslinking state of siloxane for PIS-A10 and PIS-T10 resins after postcuring at 450 °C were also detected by XPS. As shown in Figure 12, the binding energy of Si 2p peaks for PIS-T10 and PIS-A10 were centered at 103.46 and 103.41 eV, respectively, which are slightly lower than that for PIS-O10 after postcuring at 450 °C/1 h (103.59 eV). Moreover, the curve fitting results of the Si 2p peaks for PIS-A10 and PIS-T10 after postcuring at 450 °C for 1 h gave the less Q siloxy units along with more D and T siloxy units as compared with that for PIS-O10 after postcuring at 450 °C. It is suspected that the dramatic increase in *T*_g_ of PIS-O10 after postcuring at 450 °C correlated with the existence of ether groups, which promote the free radical oxidation crosslinking reaction of siloxane structure and thus further improve the *T*_g_ of the resin [35].

## 4. Conclusions

In this research, a series of PEPA-terminated oligoimides were designed and prepared by co-oligomerizing isomeric dianhydrides of a-BPDA, a-BTDA or a-ODPA with diamines mixture of APDS and TFDB, respectively. The correlation of siloxane content and dianhydride structure with the melt fluidity of the oligoimides, as well as thermal stability and mechanical properties of the cured polyimide resins were investigated. The oligoimides exhibited relatively lower melt viscosity and broader processing window accompanied with the increasing siloxane content and gave the minimum complex melt viscosity dropped in the order of PIS-T10 > PIS-A10 > PIS-O10. The PIS-O10 oligoimide derived from a-ODPA revealed excellent melt processability and melt stability with the lowest minimum dynamic melt viscosity of 0.09 Pa·s at 333 °C and the melt viscosity variation in the range of 0.69–1.63 Pa·s as isothermal treatment at 260 °C for 2 h. The cured polyimide resins displayed moderate mechanical properties except the PIS-A30 with relatively lower molecular weight. It was also found that the thermal stability of these polyimide resins was significantly affected by siloxane structure. The introduction of flexible siloxane sacrificed the thermal stability of the cured polyimide resins in some extent. However, the *T*_g_s of these polyimide resins could be significantly improved by postcuring at 400–450 °C because of the oxidative crosslinking of siloxane at elevated temperature. The PIS-O10 resin derived from a-ODPA and consisting of 10 mol% of APDS in diamines exhibited a good balance of melt processability, mechanical properties and thermal stability. The oligoimides containing siloxane structure have good potential in the fabrication of high-performance resin matrix composites by RTM.

## Figures and Tables

**Table 1:** Chemical compositions, theoretical molecular weight and GPC data of phenylethynyl-terminated oligoimides.

Samples	APDS in Diamines (mol%)	Siloxane in Oligoimide (wt%)	Theoretical *M*_n_ (g/mol)	GPC Molecular Weight		
				*M*_n_ (g/mol)	*M*_w_ (g/mol)	*M*_w_/*M*_n_
PIS-A0	0	0.00	1364	1687	2491	1.48
PIS-A10	10	1.26	1357	1529	2244	1.47
PIS-A20	20	2.56	1350	1429	2078	1.45
PIS-A30	30	3.85	1344	1356	1933	1.43
PIS-T10	10	1.24	1399	1543	2333	1.51
PIS-O10	10	1.25	1381	1398	1992	1.42

**Table 2:** The dynamic melt viscosity of PEPA-terminated oligoimides.

Samples	Minimum Melt Viscosity (Pa·s@°C)	Dynamic Viscosity (Pa·s)			Melt Viscosity Variation ^a^ @260 °C for 2 h (Pa·s)
		260 °C	270 °C	280 °C	
PIS-A0	0.55@321	20.68	5.27	2.48	21.92–69.02
PIS-A10	0.26@323	6.10	2.32	1.20	6.52–39.01
PIS-A20	0.15@323	1.82	0.82	0.53	1.89–13.89
PIS-A30	0.12@322	0.84	0.48	0.34	0.90–7.74
PIS-T10	0.58@327	7.21	2.40	1.59	7.19–34.76
PIS-O10	0.09@333	0.64	0.40	0.28	0.69–1.63

^a^ Melt viscosity variation is determined by dynamic viscosity of oligoimides held at 260 °C for 2 h.

**Table 3:** Mechanical properties of the cured polyimide resins.

Samples	Tensile Strength (MPa)	Tensile Modulus (GPa)	Elongation at Break (%)	Flexural Strength (MPa)	Flexural Modulus (GPa)
PIS-A0	70.48 ± 1.92	2.62 ± 0.12	3.90 ± 0.28	121.52 ± 2.97	4.08 ± 0.13
PIS-A10	62.82 ± 0.97	2.53 ± 0.12	3.37 ± 0.38	102.78 ± 3.36	4.05 ± 0.18
PIS-A20	62.28 ± 1.24	2.35 ± 0.11	2.77 ± 0.18	99.37 ± 2.53	3.63 ± 0.09
PIS-T10	54.69 ± 4.19	2.71 ± 0.14	2.41 ± 0.23	114.07 ± 3.18	4.62 ± 0.13
PIS-O10	58.44 ± 3.28	2.51 ± 0.15	2.43 ± 0.14	108.13 ± 5.27	3.84 ± 0.09

**Table 4:** Thermal properties of the cured polyimide resins.

Samples	Siloxane in Oligoimide (wt%)	DMA ^a^		TGA in Air ^b^		
		*E’* (°C)	tan*δ* (°C)	*T*_5_ (°C)	*T*_10_ (°C)	*R*_700_ (%)
PIS-A0	0.00	388	442	576.7	602.5	30.5
PIS-A10	1.26	330	361	560.8	593.9	32.9
PIS-A20	2.56	322	347	556.5	591.9	33.0
PIS-A30	3.85	287	323	549.2	589.4	35.1
PIS-T10	1.24	379	437	554.1	588.2	30.7
PIS-O10	1.25	303	336	539.7	577.8	27.1

^a^*E’*: onset temperature in the storage modulus curve of DMA; tan*δ*: peak temperature in the tan*δ* curve; ^b^*T*_5_ and *T*_10_: 5% and 10% weight loss temperature, respectively; *R*_700_: residual weight retention at 700 °C.

**Table 5:** The fitting data of Si 2p XPS spectra for PIS-O10 after curing at 370 °C and postcuring at different conditions.

Curing and Postcuring Conditions	D[(CH_3_)_2_SiO_2/2_]	T[CH_3_SiO_3/2_]	Q[SiO_4/2_]
	(at.%)	(at.%)	(at.%)
370 ℃/2 h	76.3	23.7	0.0
400 ℃/1 h	16.4	37.9	45.7
400 ℃/2 h	15.5	35.9	48.6
420 ℃/1 h	14.9	27.6	57.5
420 ℃/2 h	13.7	26.8	59.5
42 0℃/3 h	12.2	20.5	64.1
450 ℃/1 h	10.5	13.4	76.1

**Table 6:** Thermal properties of the polyimide resins with different dianhydride structure after postcuring at 450 °C.

Samples	DMA ^a^		TGA in Air ^b^		
	*E’* (°C)	tan*δ* (°C)	*T*_5_ (°C)	*T*_10_ (°C)	*R*_700_ (%)
PIS-A10	465	498	577.2	599.1	27.8
PIS-T10	485	>550	573.4	596.2	26.1
PIS-O10	495	>550	572.9	595.1	28.6

^a^*E’*: onset temperature in the storage modulus curve of DMA; tan*δ*: peak temperature in the tan*δ* curve; ^b^*T*_5_ and *T*_10_: 5% and 10% weight loss temperature, respectively; *R*_700_: residual weight retention at 700 °C.
